# The diagnostic value of EBV-DNA and EBV-related antibodies detection for nasopharyngeal carcinoma: a meta-analysis

**DOI:** 10.1186/s12935-021-01862-7

**Published:** 2021-03-10

**Authors:** Weixing Liu, Gui Chen, Xin Gong, Yingqi Wang, Yaoming Zheng, Xiao Liao, Wenjing Liao, Lijuan Song, Jun Xu, Xiaowen Zhang

**Affiliations:** State Key Laboratory of Respiratory Disease, Department of Otolaryngology-Head and Neck Surgery, First Affiliated Hospital, Guangzhou Medical University, #151 Yanjiangxi Road, Guangzhou, 510120 Guangdong People’s Republic of China

**Keywords:** Epstein-Barr virus, Nasopharyngeal carcinoma, EBV-DNA, EA-IgA, VCA-IgA, EBNA1-IgA, Rta-IgG, Diagnosis, Meta-analysis

## Abstract

**Background:**

Numerous individual studies have investigated the diagnostic value of EBV-DNA, EA-IgA, VCA-IgA, EBNA1-IgA and Rta-IgG detection for nasopharyngeal carcinoma (NPC), but the conclusions remain controversial. This meta-analysis aimed to determine the value of EBV-DNA, EA-IgA, VCA-IgA, EBNA1-IgA and Rta-IgG detection in the diagnosis of NPC.

**Methods:**

PROSPERO registration number: CRD42019145532. PubMed, EMBASE, Cochrane Library, and Chinese data libraries (Wanfang, CNKI, and CBM) were searched up to January 2019. The pooled sensitivity, specificity, and positive likelihood, negative likelihood, and diagnostic odds ratios were conducted in this meta-analysis. Summary receiver operating characteristic curves evaluated the test-performance global summary. Publication bias was examined by Deek’s funnel plot asymmetry test.

**Results:**

Forty-seven studies with 8382 NPC patients (NPC group) and 15,089 individuals without NPC (Control group) were included in this meta-analysis. The sensitivity, specificity, positive likelihood (+ LR), negative likelihood (-LR), DOR and AUC of **EBV-DNA** in diagnosis of NPC were: 0.76 (95% *CI* 0.73–0.77), 0.96 (95% *CI* 0.95–0.97), 14.66 (95% *CI* 9.97–21.55), 0.19 (95% *CI* 0.13–0.28), 84 (95% *CI* 50.45–139.88), 0.96 (*SE*: 0.001), and 0.55 (95% *CI* 0.54–0.57), 0.96 (95% *CI* 0.96–0.97), 12.91 (95% *CI* 9.55–17.45), 0.35 (95% *CI* 0.29–0.43), 39.57 (95% *CI* 26.44–59.23), 0.94 (*SE:* 0.002) for the **EA-IgA**, and 0.85 (95% *CI* 0.84–0.85), 0.89 (95% *CI* 0.88–0.89), 6.73 (95% *CI*5.38–8.43), 0.17 (95% *CI* 0.12–0.23), 43.03 (95% *CI* 31.51–58.76), 0.93 (*SE*: 0.007) for the **VCA-IgA**, and 0.86 (95% *CI* 0.85–0.88), 0.87 (95% *CI *0.88–0.90), 7.55 (95% *CI* 5.79–9.87), 0.16 (95% *CI* 0.13–0.19), 50.95 (95% *CI* 34.35–75.57), 0.94 (*SE:* 0.008) for the **EBNA1-IgA**, and 0.70 (95% *CI* 0.69–0.71), 0.94 (95% *CI* 0.94–0.95), 9.84 (95% *CI* 8.40–11.54), 0.25 (95% *CI* 0.21–0.31), 40.59 (95% *CI* 32.09–51.35), 0.95 (*SE:* 0.005) for the **Rta-IgG**. The EBV-DNA had larger AUC compared with other EBV-based antibodies (*P* < 0.05), while the difference between EA-IgA, VCA-IgA, EBNA1-IgA and Rta-IgG was not statistically significant (*P* > 0.05).

**Conclusions:**

EBV-DNA, VCA-IgA, EBNA1-IgA and Rta-IgG detection have high accuracy in early diagnosis NPC. In addition, EBV-DNA detection has the higher diagnosis accuracy in NPC. On the other hand, EA-IgA is suitable for the diagnosis but not NPC screening.

**Supplementary Information:**

The online version contains supplementary material available at 10.1186/s12935-021-01862-7.

## Background

Nasopharyngeal carcinoma (NPC) is the most common malignant tumor in head and neck surgery and it is highly prevalent in southern China and Southeast Asia [1]. Unfortunately, early-stage patients with NPC are asymptomatic. More than 70% newly diagnosed NPC are local-advanced or distant metastasis, and the extent of NPC at diagnosis is the most important factor affecting survival rate [2]. Despite radiotherapy and chemoradiotherapy in widespread use as the primary treatment for NPC, the overall prognosis remains poor [3]. Therefore, the use of ideal NPC early diagnosis markers is crucial. Clinical information, laboratory exams and biomedical informatics are significance component in cancer patients [4]. NPC is related to Epstein–Barr virus (EBV) infection which can promote the development of NPC [5]. The detection of specific Epstein-Barr virus DNA and antibodies are important means for the early diagnosis of NPC [6]. In addition, EBV-based antibodies detection has the advantages of rapid, convenient, and low cost. Numerous individual studies have investigated the diagnostic value of EBV-DNA, EA-IgA, VCA-IgA, EBNA1-IgA and Rta-IgG detection for nasopharyngeal carcinoma, but variable sensitivities and specificities were reported. Currently, there is no consensus which is a better test for early diagnosis of NPC. This meta-analysis aimed to determine the value of EBV-DNA, EA-IgA, VCA-IgA, EBNA1-IgA and Rta-IgG detection in the diagnosis of NPC and to provide an important basis for NPC screening and early diagnosis. This meta-analysis followed the PRISMA Diagnostic Test Accuracy reporting guidelines [7].

## Methods

PROSPERO registration number: CRD42019145532.

### Data sources and literature search strategy

Literature review was separately conducted by two investigators that queried online databases, including PubMed, EMBASE, Cochrane Library, and Chinese data libraries (WanFang, CNKI, and CBM), and the search concluded in January 2019, using the following keywords: nasopharyngeal carcinoma, Epstein-Barr virus, capsid antigen-IgA, early antigen antibody, nuclear antigen antibody, BRLF1 transcription activator IgG, EBV-DNA, EA-IgA, VCA-IgA, EBNA1-IgA and Rta-IgG.

### Study selection

#### Inclusion criteria

1. Studies that assessed the performance of EBV-DNA, EA-IgA, VCA-IgA, EBNA1-IgA and Rta-IgG detection for untreated NPC identification;
2. All patients included in the study were diagnosed using a reference test (such as needle biopsy or postoperative tissue specimens with pathological confirmation);
3. Studies that used a pre-specified threshold;
4. Studies that clearly stated the number of true positive, false positive, false negative, and true negative results in the diagnosis of NPC or these values could be calculated from the data;
5. Studies that provided a clear definition of the control sources (healthy individual or non-NPC patients);
6. In cases of multiple reports describing the same population, the most recent or most complete report was selected.

#### Exclusion criteria

1. Reported results were insufficient for construction of the 2 × 2 table;
2. Studies that failed to clearly define the control types;
3. The NPC group contained other tumors;
4. Basic research, review articles, comments, letters, case reports, abstracts in conference, responding letters and experimental animal studies.

### Study quality assessment and data extraction

Study quality assessment was conducted using the diagnostic accuracy (QUADAS) II checklist [8]. Studies considered of high quality were eligible for this meta-analysis. Data on study characteristics, the first author, year of publication, country of origin, article language, sample size, control sources (healthy individuals or non-NPC patients), detection method, sample types and cutoff value were extracted from the selected studies by one author and checked by another author. If agreement cannot be reached, a third reviewer will be consulted. Any disagreements were discussed until consensus was reached.

### Statistical analysis

Standard methods recommended for meta-analyses of diagnostic test evaluations were used to perform this meta-analysis [9]. Review Manager version 5.3, Meta-DiSc statistical software version 1.4 and Stata version 14.0 (STATA Corporation, College Station, TX, USA) were used in this meta-analysis. The Cochrane *Q* test and inconsistency index (*I*^*2*^) were used to estimate the heterogeneity within studies [10]. Heterogeneity was considered statistically significant when *P* < 0.05 or *I*^*2*^ > 50%. If statistically significant heterogeneity existed, meta-analysis was performed using the random effects model, otherwise, a fixed effect model was used.

The accuracy indexes of EBV-DNA, EA-IgA, VCA-IgA, EBNA1-IgA and Rta-IgG was pooled by meta-analysis, such as sensitivity, specificity, positive likelihood ratio (PLR), negative likelihood ratio (NLR), diagnostic odds ratio (DOR) and AUC. The likelihood ratios (PLR and NLR) are clinically meaningful for the measurement of diagnostic accuracy; PLR > 10 and NLR < 0.1 are considered high [11]. The DOR is a single indicator of test accuracy that combines the data from sensitivity and specificity into a single metric. The summary receiver operating characteristic (SROC) curve was used to evaluate the global summary of test performance.

Sensitivity analyses were performed to explore the sources of heterogeneity of the included studies by removing each included study consecutively. The heterogeneity was investigated by meta-regression according to different covariates, including publication year (Year ≥ 2011 or < 2011), NPC size (NPC ≥ 100 or NPC < 100), control sources (Control sources from healthy serum or from healthy persons and non-NPC patients), detection method, and article language (English or Chinese). Publication bias was examined by Deek’s funnel plot asymmetry test. All *P* values were two sides and *P* < *0.05* was regarded as statistically significant.

## Results

### Article search and study quality

**Fig. 1:**
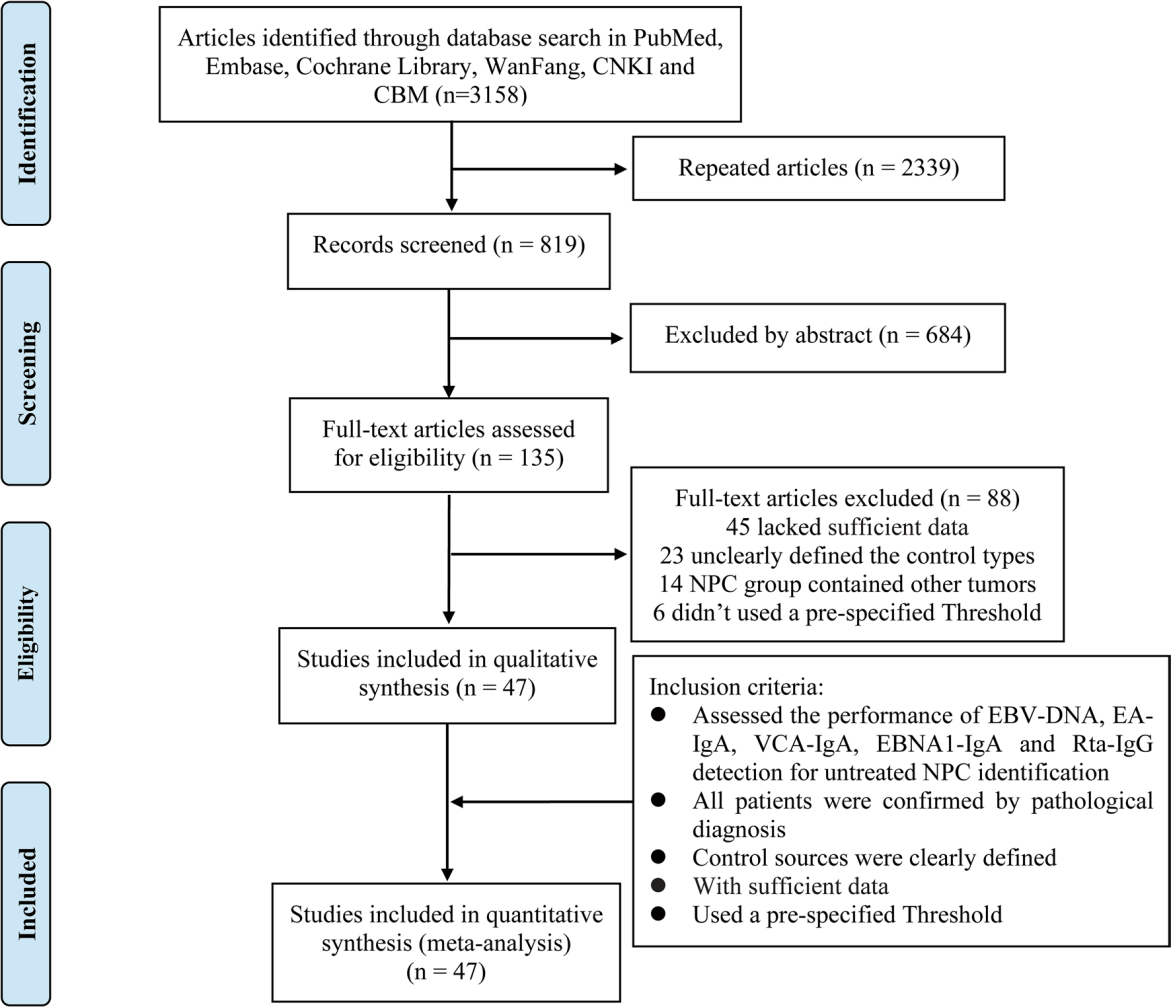
Flowchart of study selection

**Fig. 2:**
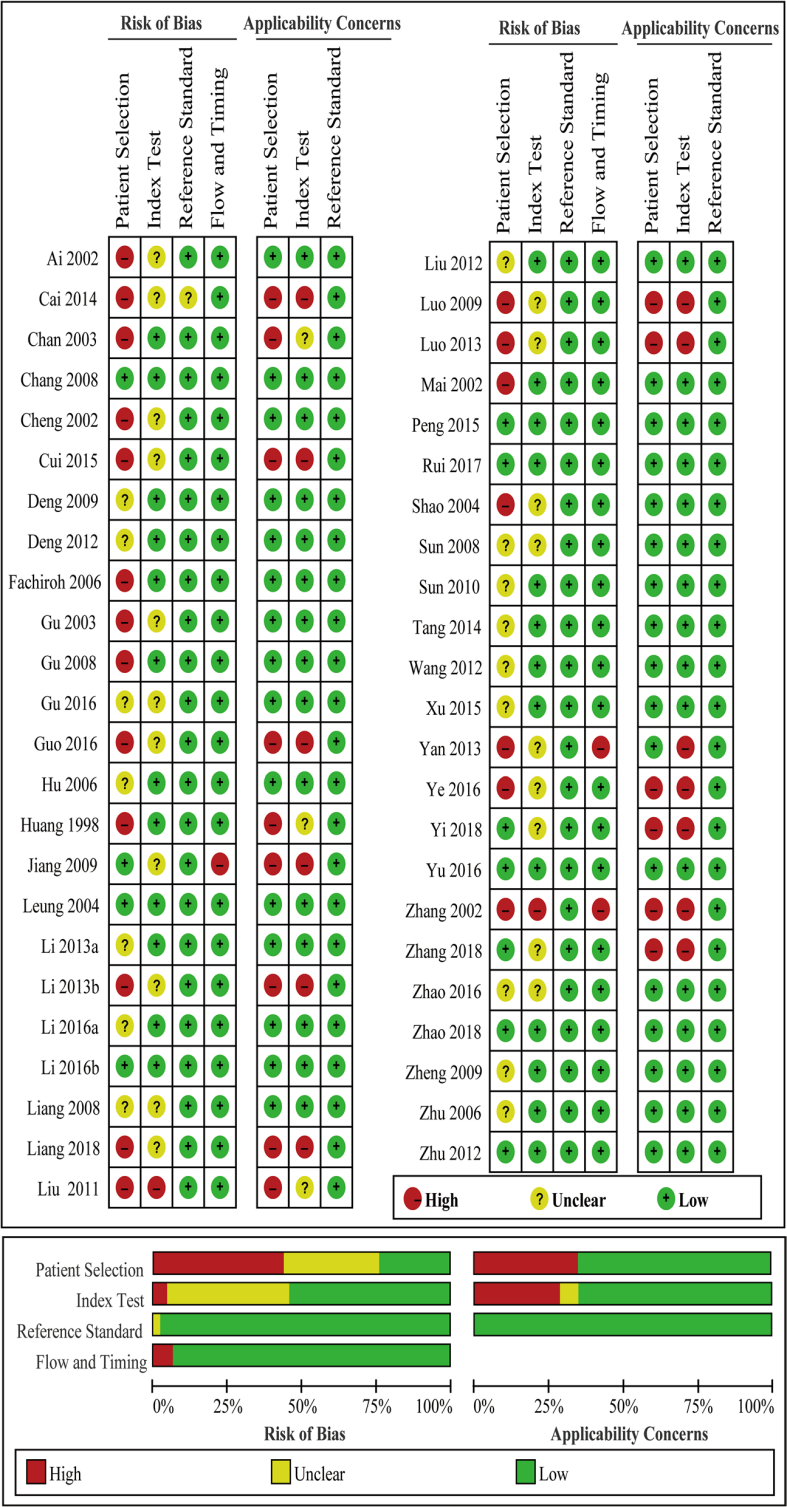
Assessment of the reporting quality of the included studies using the QUADAS II checklist

In this meta-analysis, 47 publications on the role of EBV-DNA, EA-IgA, VCA-IgA, EBNA1-IgA and Rta-IgG concentrations in the diagnosis of NPC that met the criteria for inclusion were included in the analysis [12–58]. Figure 1 shows a flowchart of the study selection process. The 47 studies included 8382 patients with NPC (NPC group) and 15,089 patients without NPC (Control group). The main features of enrolled studies are summarized in Table 1. Article quality was judged in terms of the QUADAS II recommendations. The proportions of studies with low, high, or unclear risk of bias and applicability concerns are displayed in Fig. 2.

**Table 1:** Summary data from the 47 included studies

Study ID	Area	Language	NPC	Con	Method
Huang [12]	Fu Jian	Chinese	63	51	VCA-IgA, EA-IgA
Mai [13]	Guang Dong	English	66	58	EBV-DNA, VCA-IgA
Cheng [14]	Guang Dong	Chinese	121	332	VCA- IgA, EBNA1-IgA
Zhang [15]	Guang Dong	Chinese	266	347	VCA-IgA, EA-IgA
Gu [16]	Guang Dong	English	57	58	EBNA1-lgA
Chan [17]	Hong Kong	English	55	163	EBV-DNA, VCA- IgA, EA-IgA, EBNA1-lgA
Shao [18]	Guang Dong	English	150	75	EBV-DNA
Leung [19]	Hong Kong	English	139	178	EBV-DNA, VCA-IgA
Hu [20]	Guang Dong	Chinese	85	132	EBNA1-lgA
Fachiroh [21]	Indonesia	English	151	254	EBNA1-lgA
Zhu [22]	Guang Xi	Chinese	274	353	VCA-IgA, Rta-IgG
Liang [23]	Guang Dong	Chinese	195	188	EBNA1-lgA
Sun [24]	Hu Nan	Chinese	68	90	EBV-DNA, VCA-IgA
Chang [25]	Tai Wan	English	156	264	EBV-DNA, VCA-IgA
Gu [26]	Guang Dong	English	135	130	VCA- IgA, EBNA1-lgA
Zheng [27]	Guang Xi	Chinese	211	413	Rta-IgG
Luo [28]	Guang Dong	Chinese	160	76	EBV-DNA, VCA- IgA, EA-IgA
Jiang [29]	Guang Dong	Chinese	81	89	VCA- IgA, EA-IgA, EBNA1-lgA
Deng [30]	Guang Dong	Chinese	93	185	VCA- IgA, EBNA1-lgA
Kong [31]	Shang Dong	Chinese	56	60	EBV-DNA
Sun [32]	Hu Nan	Chinese	62	62	EBV-DNA, VCA-IgA
Liu [33]	Guang Dong	English	191	337	VCA- IgA, EA-IgA, EBNA1-lgA
Liu [34]	Hu Bei	Chinese	50	50	EBV-DNA
Zhu [35]	Jiang Su	Chinese	168	60	EBV-DNA, VCA-IgA
Wang [36]	Shang Hai	Chinese	206	248	VCA-IgA, EBNA1-IgA, Rta-lgG
Ai [37]	Si Chuan	English	100	60	VCA-IgA, EBNA1-lgA, Rta-IgG
Deng [38]	Guang Dong	Chinese	124	173	VCA-IgA, EBNA1-lgA
Li [39]	Guang Dong	Chinese	145	140	EBV-DNA
Li [40]	Fu Jian	Chinese	449	82	VCA-IgA, EA-IgA, Rta-IgG
Luo [41]	Guang Zhou	Chinese	131	200	EBV-DNA, VCA-lgA, EA-IgA, Rta-IgG
Yan [42]	Bei Jing	Chinese	50	51	VCA-IgA, EA-IgA
Tang [43]	Guang Xi	Chinese	150	150	Rta-IgG
Cai [44]	Guang Xi	English	211	413	VCA-IgA, EA-IgA, EBNA1-lgA, Rta-IgG
Peng [45]	Guang Dong	English	310	218	VCA-IgA
Xu [46]	Guang Dong	Chinese	75	100	VCA-IgA, Rta-IgG
Cui [47]	Shan Xi	English	64	120	VCA-IgA, EA-IgA, Rta-IgG
Ye [48]	Fu Jian	Chinese	160	299	EBV-DNA, VCA-IgA, EA-IgA, Rta-IgG
Li [49]	Guang Dong	English	208	198	EBV-DNA, VCA-IgA
Yu [50]	Guang Dong	Chinese	152	675	EBV-DNA, VCA-IgA, EBNA1-lgA
Li [51]	Shang Hai	English	56	90	EBV-DNA, VCA-IgA, Rta-IgG, EA-IgG
Zhao [52]	Guang Xi	Chinese	89	120	Rta-IgG
Gu [53]	Guang Dong	Chinese	60	60	VCA- IgA, EBNA1-lgA
Guo [54]	Fu Jian	Chinese	2155	6957	VCA-IgA, EA-IgA, Rta-IgG
Rui [55]	Guang Dong	English	200	200	VCA-IgA, EBNA1-IgA
Zhao [56]	Guang Dong	Chinese	80	80	EBV-DNA
Yi [57]	Fu Jian	Chinese	96	250	VCA-IgA, EA-IgA, Rta-IgG
Zhang [58]	Hu Nan	Chinese	58	200	VCA-IgA, EA-IgA, Rta-IgG

### Heterogeneity investigation

The inconsistency index (EBV-DNA: *I*^*2*^ = 77.5%, *P* < 0.001; EA-IgA:77.3%, *P* < 0.001; VCA-IgA: 87.0%, *P* < 0.001; EBNA1-IgA:78.9%, *P* < 0.001; Rta-IgG: 60.5%, *P* < 0.001) indicated significant heterogeneity among the studies. The result showed that there was no threshold effect in the pooled analysis of EBV-DNA (*P* < 0.001), EA-IgA (*P* < 0.001), VCA-IgA (*P* < 0.001), EBNA1-IgA (*P* < 0.001) and Rta-IgG (*P* < 0.001).

### Diagnostic accuracy

**Fig. 3:**
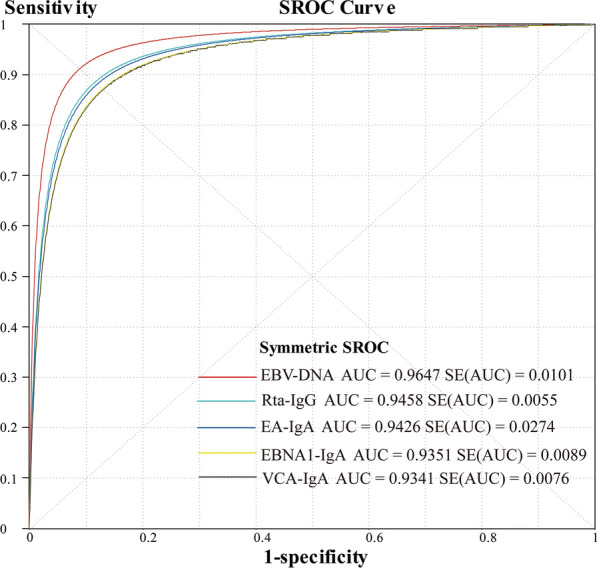
The summary receiver operator curve of EBV-DNA, EA-IgA, VCA-IgA, EBNA1-IgA and Rta-IgG detection for NPC

The pooled sensitivity, specificity, PLR, NLR, DOR and AUC for the value of EBV-DNA, EA-IgA, VCA-IgA, EBNA1-IgA and Rta-IgG in the diagnosis of NPC are displayed in Table 2 and the the diagnostic characteristics of included studies are in Tables 3, 4, 5, 6, 7. The EBV-DNA had larger areas under the summary receiver operator curve when compared with EA-IgA, VCA-IgA, EBNA1-IgA and Rta-IgG (*P* < 0.05), while EA-IgA, VCA-IgA, EBNA1-IgA and Rta-IgG were no statistically different from each other (*P* > 0.05) in Table 8. The summary receiver operator curve of EBV-DNA, EA-IgA, VCA-IgA, EBNA1-IgA and Rta-IgG detection for NPC were showed in Fig. 3. Additional file 1 showed the Forest plots of sensitivity, specificity, PLR, NLR, DOR for acoustic analysis of EBV-DNA, EA-IgA, VCA-IgA, EBNA1-IgA and Rta-IgG.

**Table 2:** The pooled result of EBV-DNA, EA-IgA, VCA-IgA, EBNA1-IgA and Rta-IgG in the diagnosis of NPC

Method	Pooled sensitivity (95% CI)	Pooled specificity (95% CI)	Pooled PLR (95% CI)	Pooled NLR (95% CI)	Pooled DOR (95% CI)	AUC (SE)
EBV-DNA	0.76	0.96	14.66	0.19	84.00	0.96
	0.73–0.77	0.95–0.97	9.97–21.55	0.13–0.28	50.45–139.88	0.0011
EA-IgA	0.55	0.96	12.91	0.35	39.57	0.94
	0.54–0.57	0.96–0.97	9.55–17.45	0.29–0.43	26.44–59.23	0.00274
VCA-IgA	0.85	0.89	6.73	0.17	43.03	0.93
	0.84–0.85	0.88–0.89	5.38–8.43	0.12–0.23	31.51–58.76	0.0076
EBNA1-IgA	0.86	0.87	7.55	0.16	50.95	0.94
	0.85–0.88	0.88–0.90	5.79–9.87	0.13–0.19	34.35–75.57	0.0089
Rta-IgG	0.70	0.94	9.84	0.25	40.59	0.95
	0.69–0.7149	0.94–0.95	8.40–11.54	0.21–0.31	32.09–51.35	0.0052

**Table 3:** The diagnostic characteristics of included studies on EBV-DNA

Study ID	TP	FP	FN	TN	Sensitive (95% CI)	Specificity (95% CI)
Mai [13]	56	6	10	52	0.85 (0.74–0.93)	0.90 (0.79–0.96)
Chan [17]	31	3	24	160	0.56 (0.42–0.70)	0.98 (0.95–0.99)
Shao [18]	138	9	12	66	0.92 (0.86–0.95)	0.88 (0.78–0.94)
Leung [19]	132	4	7	174	0.95 (0.90–0.98)	0.98 (0.94–0.99)
Sun [24]	65	6	3	84	0.96 (0.88–0.99)	0.93 (0.86–0.98)
Chang [25]	127	9	29	255	0.81 (0.74–0.87)	0.97 (0.94–0.98)
Luo [28]	110	9	50	67	0.69 (0.61–0.75)	0.88 (0.79–0.94)
Sun [32]	59	4	3	58	0.95 (0.86–0.99)	0.94 (0.84–0.98)
Liu [34]	46	4	4	46	0.92 (0.80–0.98)	0.92 (0.81–0.98)
Zhu [35]	58	2	110	58	0.35 (0.27–0.42)	0.97 (0.89–0.99)
Li [39]	136	10	9	130	0.94 (0.89–0.97)	0.93 (0.87–0.97)
Luo [41]	85	6	46	194	0.65 (0.56–0.73)	0.97 (0.94–0.99)
Ye [48]	94	7	66	292	0.59 (0.51–0.67)	0.98 (0.95–0.99)
Li [49]	149	10	59	188	0.72 (0.65–0.78)	0.95 (0.91–0.98)
Yu [50]	123	3	29	672	0.81 (0.74–0.87)	0.99 (0.98–0.99)
Li [51]	37	3	19	87	0.66 (0.52–0.78)	0.97 (0.91–0.99)
Zhao [56]	72	16	8	64	0.90 (0.81–0.97)	0.80 (0.70–0.88)

**Table 4:** The diagnostic characteristics of included studies on EA-IgA

Study ID	TP	FP	FN	TN	Sensitive (95% CI)	Specificity (95% CI)
Huang [12]	36	1	27	50	0.57 (0.44–0.70)	0.98 (0.90–1.00)
Zhang [15]	239	41	27	306	0.90 (0.86–0.93)	0.88 (0.84–0.91)
Chan [17]	40	5	15	158	0.73 (0.59–0.84)	0.97 (0.93–0.99)
Luo [28]	120	4	40	72	0.75 (0.68–0.82)	0.95 (0.87–0.99)
Jiang [29]	53	5	28	84	0.65 (0.54–0.76)	0.94 (0.87–0.98)
Liu [33]	89	17	102	320	0.47 (0.39–0.54)	0.95 (0.92–0.97)
Luo [41]	98	1	33	199	0.75 (0.67–0.82)	0.99 (0.97–1.00)
Li [40]	210	6	239	76	0.47 (0.42–0.52)	0.93 (0.85–0.97)
Yan [42]	16	2	35	48	0.31 (0.19–0.46)	0.96 (0.86–0.99)
Cai [44]	188	3	23	200	0.89 (0.84–0.93)	0.99 (0.96–0.99)
Cui [47]	46	4	18	116	0.72 (0.59–0.82)	0.98 (0.92–0.99)
Ye [48]	94	12	66	287	0.59 (0.51–0.67)	0.96 (0.93–0.98)
Guo [54]	1004	213	1151	6744	0.47 (0.45–0.49)	0.97 (0.97–0.97)
Yi [57]	47	10	49	240	0.49 (0.39–0.59)	0.96 (0.93–0.98)
Zhang [58]	42	23	16	177	0.72 (0.59–0.83)	0.89 (0.83–0.93)

**Table 5:** The diagnostic characteristics of included studies on VCA-IgA

Study ID	TP	FP	FN	TN	Sensitive (95% CI)	Specificity (95% CI)
Huang [12]	62	6	1	55	0.98 (0.92–1.00)	0.90 (0.80–0.96)
Mai [13]	53	6	13	52	0.80 (0.69–0.89)	0.90 (0.79–0.96)
Cheng [14]	112	43	9	289	0.93 (0.86–0.97)	0.87 (0.83–0.91)
Zhang [15]	241	19	25	328	0.97 (0.86–0.94)	0.95 (0.91–0.97)
Chan [17]	51	60	4	98	0.94 (0.82–0.98)	0.62 (0.54–0.70)
Leung [19]	112	8	27	170	0.87 (0.73–0.87)	0.96 (0.91–0.98)
Zhu [22]	248	53	26	300	0.91 (0.86–0.94)	0.85 (0.80–0.89)
Sun [24]	63	45	5	45	0.93 (0.84–0.98)	0.50 (0.39–0.61)
Chang [25]	134	36	22	228	0.86 (0.79–0.91)	0.86 (0.82–0.90)
Gu [26]	124	37	15	93	0.89 (0.83–0.94)	0.71 (0.63–0.79)
Luo [28]	144	8	16	68	0.90 (0.84–0.94)	0.90 (0.80–0.95)
Jiang [29]	77	9	4	80	0.95 (0.88–0.99)	0.90 (0.81–0.95)
Deng [30]	81	12	12	173	0.87 (0.79–0.93)	0.94 (0.89–0.97)
Sun [32]	63	45	5	45	0.93 (0.84–0.98)	0.50 (0.39–0.61)
Liu [33]	174	65	17	272	0.91 (0.86–0.95)	0.81 (0.76–0.85)
Zhu [35]	105	2	63	28	0.63 (0.55–0.70)	0.93 (0.78–0.99)
Wang [36]	179	11	27	237	0.87 (0.81–0.91)	0.96 (0.92–0.98)
Ai [37]	43	4	57	56	0.43 (0.33–0.53)	0.93 (0.84–0.98)
Deng [38]	94	15	30	158	0.76 (0.67–0.83)	0.91 (0.86–0.95)
Luo [41]	122	26	9	305	0.93 (0.87–0.97)	0.92 (0.89–0.95)
Li [40]	397	18	52	64	0.88 (0.85–0.91)	0.78 (0.68–0.86)
Yan [42]	39	3	12	47	0.77 (0.63–0.87)	0.94 (0.84–0.99)
Cai [44]	207	35	14	168	0.94 (0.90–0.97)	0.83 (0.77–0.88)
Peng [45]	163	16	147	202	0.53 (0.47–0.58)	0.93 (0.88–0.96)
Xu [46]	67	16	8	84	0.89 (0.80–0.95)	0.84 (0.75–0.91)
Cui [47]	51	6	13	114	0.80 (0.68–0.89)	0.95 (0.89–0.98)
Ye [48]	151	49	9	250	0.94 (0.90–0.97)	0.84 (0.79–0.88)
Li [49]	176	13	32	185	0.85 (0.79–0.89)	0.93 (0.89–0.96)
Yu [50]	68	56	84	619	0.45 (0.37–0.53)	0.91 (0.89–0.93)
Li [51]	44	8	12	81	0.79 (0.67–0.88)	0.91 (0.83–0.96)
Gu [53]	20	11	40	49	0.33 (0.22–0.47)	0.82 (0.70–0.91)
Guo [54]	1937	710	218	6247	0.90 (0.89–0.91)	0.90 (0.89–0.91)
Rui [55]	176	29	24	171	0.88 (0.83–0.92)	0.86 (0.80–0.90)
Yi [57]	85	28	11	222	0.89 (0.80–0.94)	0.89 (0.84–0.92)
Zhang [58]	36	20	12	180	0.75 (0.60–0.86)	0.90 (0.85–0.94)

**Table 6:** The diagnostic characteristics of included studies on EBNA1-IgA

Study ID	TP	FP	FN	TN	Sensitive (95% CI)	Specificity (95% CI)
Cheng [14]	103	50	18	285	0.85 (0.78–0.91)	0.85 (0.80–0.89)
Gu [16]	52	7	6	51	0.90 (0.79–0.96)	0.88 (0.78–0.95)
Chan [17]	46	22	9	141	0.84 (0.71–0.92)	0.87 (0.80–0.91)
Hu [20]	69	25	16	107	0.81 (0.71–0.89)	0.81 (0.73–0.87)
Fachiroh [21]	134	51	17	203	0.89 (0.83–0.93)	0.80 (0.75–0.85)
Liang [23]	166	28	29	160	0.85 (0.79–0.90)	0.85 (0.79–0.90)
Gu [26]	108	26	27	104	0.80 (0.72–0.86)	0.80 (0.72–0.87)
Deng [30]	83	10	10	175	0.89 (0.81–0.95)	0.95 (0.90–0.97)
Liu [33]	177	48	14	289	0.93 (0.88–0.96)	0.86 (0.81–0.89)
Wang [36]	94	11	12	238	0.89 (0.81–0.94)	0.96 (0.92–0.98)
Ai [37]	85	12	15	48	0.85 (0.77–0.91)	0.80 (0.68–0.89)
Deng [38]	137	9	31	77	0.82 (0.75–0.87)	0.90 (0.81–0.95)
Cai [44]	184	32	27	171	0.87 (0.82–0.91)	0.84 (0.79–0.89)
Yu [50]	120	21	32	654	0.79 (0.72–0.85)	0.97 (0.95–0.98)
Gu [53]	53	2	7	58	0.88 (0.77–0.95)	0.97 (0.89–1.00)
Rui [55]	188	15	12	185	0.94 (0.90–0.97)	0.93 (0.88–0.96)

**Table 7:** The diagnostic characteristics of included studies on Rta-IgG

Study ID	TP	FP	FN	TN	Sensitive (95% CI)	Specificity (95% CI)
Zhu [22]	225	29	49	324	0.82 (0.77–0.87)	0.92 (0.88–0.94)
Zheng [27]	191	41	20	372	0.91 (0.86–0.94)	0.90 (0.87–0.93)
Wang [36]	132	21	74	228	0.64 (0.57–0.71)	0.92 (0.87–0.95)
Ai [37]	77	5	23	55	0.77 (0.68–0.85)	0.92 (0.82–0.97)
Luo [41]	102	15	29	185	0.78 (0.70–0.85)	0.93 (0.88–0.96)
Li [40]	335	6	114	76	0.75 (0.70–0.79)	0.93 (0.85–0.97)
Tang [43]	134	16	17	133	0.89 (0.83–0.93)	0.89 (0.83–0.94)
Cai [44]	191	30	20	173	0.91 (0.86–0.94)	0.85 (0.80–0.90)
Xu [46]	61	7	14	93	0.81 (0.71–0.89)	0.93 (0.86–0.97)
Cui [47]	48	4	16	116	0.75 (0.63–0.85)	0.97 (0.92–0.99)
Ye [48]	122	17	38	282	0.76 (0.69–0.83)	0.94 (0.91–0.97)
Li [51]	43	7	13	83	0.77 (0.64–0.87)	0.92 (0.85–0.97)
Zhao [52]	67	6	21	124	0.76 (0.66–0.85)	0.95 (0.90–0.98)
Guo [54]	1363	352	792	6605	0.63 (0.61–0.65)	0.95 (0.94–0.95)
Yi [57]	63	12	33	238	0.66 (0.55–0.75)	0.95 (0.92–0.98)
Zhang [58]	42	23	16	177	0.72 (0.59–0.83)	0.89 (0.83–0.93)

**Table 8:** The Z test of EBV-DNA, EA-IgA, VCA-IgA, EBNA1-IgA and Rta-IgG in the diagnosis of NPC

Method	Z	P
EBV-DNA VS VCA-IgA	3.61	< 0.001
EBV-DNA VS EA-IgA	6.52	< 0.001
EBV-DNA VS EBNA1-IgA	2.98	0.003
EBV-DNA VS Rta-IgG	2.69	0.007
VCA-IgA VS EA-IgA	− 1.05	0.293
VCA-IgA VS EBNA1-IgA	− 0.09	0.932
VCA-IgA VS Rta-IgG	− 1.46	0.146
EA-IgA VS EBNA1-IgA	0.81	0.421
EA-IgA VS Rta-IgG	− 0.84	0.404
EBNA1-IgA VS Rta-IgG	− 1.20	0.229

### Sensitivity analysis and meta-regression

The sensitivity analysis showed that the results were not affected by the exclusion of any individual trial. As meta-regression result indicated that publication year, NPC or control size, control sources, detection method, cutoff value, and article language are not the DOR heterogeneity of EBV-DNA, VCA-IgA, EBNA1-IgA and Rta-IgG, whereas detection method was possible DOR heterogeneity sources for the EA-IgA (*P* < 0.0095).

### Publication bias

Publication bias was judged by Deek’s funnel plot asymmetry test, and the statistical results revealed no significant publication bias among studies about EBV-DNA (*P* = 0.14), EA-IgA (*P* = 0.26), EBNA1-IgA (*P* = 0.56) and Rta-IgG (*P* = 0.16), other than VCA-IgA (*P* = 0.03) (Additional file 1).

## Discussion

EBV infection plays a critical role in the progression of nasopharyngeal carcinoma, as body can produce lots of EBV-related antigens at the early stage, which can be used for NPC screening and EBV-DNA, EA-IgA, VCA-IgA and EBNA1-IgA are usually involved [59, 60]. EBV-DNA in circulation may be released from cancer cells during the process of apoptosis or generated from viral replication and different EBV antigens are expressed at different stages of infection [61]. Circulating EBV-DNA has been shown to correlate with the stage of NPC, recurrence rate and screening for NPC [62]. In this study, a meta-analysis was conducted to assess the diagnostic significance of five EBV-based markers for patients with NPC. This study showed that the EBV-DNA, EA-IgA, VCA-IgA, EBNA1-IgA and Rta-IgG detection were effective method for NPC diagnosis.

Previous meta-analyses have been published on the value of some EBV-based markers in the detection of NPC. For the EBV-DNA, Han et al.conducted a meta-analysis based on 18 studies involving 1492 NPC cases and 2461 health controls in Asians, in which the pooled sensitivity and specificity of EBV-DNA detection for NPC were 0.73 (95% *CI* 0.71–0.75) and 0.89 (95% *CI* 0.88–0.90) [63]. Furthermore, Han et al.found that the accuracy of NPC detection was lower by serum (0.81) than that by plasma (0.86), with SROCs being 0.91 and 0.97, respectively. The heterogeneity across studies showed significant difference in the Han’s study, and Han et al.did not evaluate the threshold effect and publication bias [63]. The Han’s study should also perform sensitivity analysis and meta-regression to explore the sources of heterogeneity. Li et al. conducted another important meta-analysis on the diagnosis value of VCA-IgA detection for NPC based on 4671 patients with NPC and 7663 patients without NPC [64], which was correlated with higher pooled sensitivity 0.91 (95% *CI* 0.90–0.92) and specificity 0.92 (95% *CI *0.92–0.93), with SROC 0.98. But the Li’s study existed language bias and publication bias. For the Rta-IgG, Cui et al.pooled 17 studies involving 2658 NPC patients, and the results pointed out that the sensitivity of Rta-IgG for detecting NPC was 90.83 (95% *CI* 0.78–0.87), the specificity was 0.92 (95% *CI* 0.90–0.93) [65]. Threshold effect, publication bias as well as complicated control types presented in the Cui’s study, which may contribute to heterogeneity and affect the accuracy of pooled results. Additionally, previous meta-analyses could not reach a conclusive result as to the most favorable choice for NPC diagnosis.

To our knowledge, this is the first meta-analysis to determine the usefulness of EA-IgA and EBNA1-IgA and compare the accuracy EBV-DNA, EA-IgA, VCA-IgA, EBNA1-IgA and Rta-IgA in diagnosis of NPC. In this study, the highest sensitivity was EBNA1-IgA (0.86), and the specificity of EBV-DNA (0.96) and EA-IgA (0.96) were highest. Besides, the sensitivity of EA-IgA (0.55) was lowest, and screening for NPC using only EA-IgA may lead to misdiagnosis, but the specificity was high, which indicated that EA-IgA was suitable for the diagnosis but not screening of NPC. EA-IgA or EBV-DNA detection combined with other indicators may also improve the sensitivity and specificity for the serological diagnosis of NPC [44, 51]. The likelihood ratios (PLR and NLR) calculated from the sensitivity and specificity indicate the discriminatory properties of negative and positive test results. The pooled PLR of EBV-DNA, VCA-IgA and EA-IgA were above 5 and NLR below 0.2, which given strong diagnosis evidence especially for EBV-DNA and EA-IgA with PLR above 10 [11]. Furthermore, a summary receiver operating characteristic (SROC) curve was also conducted to describe the relationship between sensitivity and specificity. AUC can summarize the inherent capacity of a test to discriminate the participant with disease from those without it [66, 67]. The AUC of EBV-DNA and other antibodies were more than 90%, indicating a very high level of overall accuracy. The EBV-DNA (AUC = 0.96) had slightly larger AUC compared with EA-IgA, VCA-IgA, EBNA1-IgA and Rta-IgG (*P* < 0.05). Additionally, the pooled DOR of EBV-DNA (84.00) that differed 33.05–44.43 was higher than other EBV-based antibodies. These results indicated that EBV-DNA detection had higher accuracy in diagnosis of NPC. In addition, a meta-analysis included 8128 NPC cases showed that pre-EBV-DNA levels can also be a prognostic indictor for patients with NPC [68]. A recent prospective screening study involving 20,174 participants showed that plasma EBV-DNA detection was useful in screening for early asymptomatic nasopharyngeal carcinoma screening, with 97.1% sensitivity and 98.6% specificity [2]. But only 309/1112 had detectable Epstein-Barr virus DNA in plasma at baseline and at follow-up, and 35 patients had confirmed nasopharyngeal carcinoma. In Nicholls’ study [15], seventy-eight NPC patients (15.1%) were plasma EBV-DNA negative who had similar 5-year overall survival and cancer-specific survival to those EBV-DNA positive counterparts by stage. If only plasma EBV-DNA was used as the population screening tool, 60.0%, 23.0%, 14.5% and 5.0% of stage I, II, III and IVA NPC may be missed. The golden standard for cancer prognosis is pathological examination following the complicated and painful procedures of biopsy, which may not be feasible by some patients [69]. In addition, endoscopic, computed tomography (CT) and Magnetic resonance imaging (MRI) has been the imaging modality of choice for cancer diagnosis and staging [70, 71]. In practice, due to the current limitations of a single serum index, multiple assays (nasal endoscopy, CT and MRI) and biopsies [70, 72], it is still necessary develop methods to increase NPC early diagnostic rate.

Several strengths of present meta-analysis should be highlighted. This study compered five EBV-related diagnostic markers for NPC with comprehensive calculations of their diagnostic performance, shedding light on the value of these tests in clinical settings. In the context of current availability of studies on EBV-DNA and immunoglobin antibody tests in the literature, this meta-analysis covers a large sample size pooled from rigorously included studies, and the results were stable. However, this meta-analysis should be interpreted with caution due to certain limitations. First, there was large heterogeneity among the included studies with differences in characteristics of the study and participants. The inability to obtain raw data on patient age and gender may have led to the heterogeneity and hindered a more detailed analysis. Second, most of the included populations were Chinese, which could lead to population selection bias and should not allow for generalization to other ethnicity groups, rendering further research needed. Third, technical methods for testing of EBV-related markers vary across different studies, including the inconsistent cut-off values and different antigen sets used despite of a same generic name. Use of enzyme-linked immunosorbent assay or immunofluorescence assay may have contributed to the different results [73]. Finally, most of the studies included NPC cases and controls in a single institution or from a same geographic region, which could have influenced the results of the study. Therefore, a larger, prospective, randomized and multicentered clinical trial should been done to evaluate the diagnostic value of EBV-based tools in the diagnosis of NPC.

## Conclusions

Notwithstanding heterogeneity of currently available data, the studies included in our analysis are of a large sample size and high-quality, thus providing a considerable power. EBV-DNA, VCA-IgA, EBNA1-IgA and Rta-IgG detection have high accuracy in early diagnosis NPC and can improve the effectiveness of screening. In addition, EBV-DNA detection has the higher diagnosis accuracy in NPC. On the other hand, EA-IgA is suitable for the diagnosis but not NPC screening. Further well-designed clinical trials need to be carried out in order to improve early diagnosis rate.

## Supplementary Information

**Additional file 1:** Forest plots of sensitivity, specificity, PLR, NLR, DOR for acoustic analysis of EBV-DNA, EA-IgA, VCA-IgA, EBNA1-IgA and Rta-IgG, and Funnel plots for publication bias test.

## Data Availability

The datasets used during the current study are available from the corresponding author on reasonable request.

## Abbreviations

NPC: Nasopharyngeal carcinoma
CI: Confidence interval
PLR: Positive likelihood ratio
NLR: Negative likelihood ratio
DOR: Diagnostic odds ratio
AUC: Area under the Curve
SROC: Summary receiver operating characteristic
