# Supplementary material for: The diagnostic value of EBV-DNA and EBV-related antibodies detection for nasopharyngeal carcinoma: a meta-analysis
Source: Cancer Cell Int. 2021 Mar 10;21:164. doi: 10.1186/s12935-021-01862-7 (PMC7944913; doi:10.1186/s12935-021-01862-7)
Supplement: Supplementary file 1 — Additional file 1: Forest plots of sensitivity, specificity, PLR, NLR, DOR for acoustic analysis of EBV-DNA, EA-IgA, VCA-IgA, EBNA1-IgA and Rta-IgG, and Funnel plots for publication bias test. [file 12935_2021_1862_MOESM1_ESM.docx]

**Figure legends**

**Fig. 1.1** Forest plots of sensitivity estimates for acoustic analysis of EBV-DNA in the diagnosis of NPC.

**Fig. 1.2** Forest plots of specificity estimates for acoustic analysis of EBV-DNA in the diagnosis of NPC.

**Fig. 1.3** Forest plots of positive likelihood estimates for acoustic analysis of EBV-DNA in the diagnosis of NPC.

**Fig. 1.4** Forest plots of negative likelihood estimates for acoustic analysis of EBV-DNA in the diagnosis of NPC.

**Fig. 1.5** Forest plots of DOR estimates for acoustic analysis of EBV-DNA in the diagnosis of NPC.

**Fig. 1.6** Summary receiver operating characteristic curve of EBV-DNA in the diagnosis of NPC.

**Fig. 1.7** Deek’s funnel plot of EBV-DNA.

**Fig. 2.1** Forest plots of sensitivity estimates for acoustic analysis of EA-IgA in the diagnosis of NPC.

**Fig. 2.2** Forest plots of specificity estimates for acoustic analysis of EA-IgA in the diagnosis of NPC.

**Fig. 2.3** Forest plots of positive likelihood estimates for acoustic analysis of EA-IgA in the diagnosis of NPC.

**Fig. 2.4** Forest plots of negative likelihood estimates for acoustic analysis of EA-IgA in the diagnosis of NPC.

**Fig. 2.5** Forest plots of DOR estimates for acoustic analysis of EA-IgA in the diagnosis of NPC.

**Fig. 2.6** Summary receiver operating characteristic curve of EA-IgA in the diagnosis of NPC.

**Fig. 2.7** Deek’s funnel plot of EA-IgA.

**Fig. 3.1** Forest plots of sensitivity estimates for acoustic analysis of VCA-IgA in the diagnosis of NPC.

**Fig. 3.2** Forest plots of specificity estimates for acoustic analysis of VCA-IgA in the diagnosis of NPC.

**Fig. 3.3** Forest plots of positive likelihood estimates for acoustic analysis of VCA-IgA in the diagnosis of NPC.

**Fig. 3.4** Forest plots of negative likelihood estimates for acoustic analysis of VCA-IgA in the diagnosis of NPC.

**Fig. 3.5** Forest plots of DOR estimates for acoustic analysis of VCA-IgA in the diagnosis of NPC.

**Fig. 3.6** Summary receiver operating characteristic curve of VCA-IgA in the diagnosis of NPC.

**Fig. 3.7** Deek’s funnel plot of VCA-IgA.

**Fig. 4.1** Forest plots of sensitivity estimates for acoustic analysis of EBNA1-IgA in the diagnosis of NPC.

**Fig. 4.2** Forest plots of specificity estimates for acoustic analysis of EBNA1-IgA in the diagnosis of NPC.

**Fig. 4.3** Forest plots of positive likelihood estimates for acoustic analysis of EBNA1-IgA in the diagnosis of NPC.

**Fig. 4.4** Forest plots of negative likelihood estimates for acoustic analysis of EBNA1-IgA in the diagnosis of NPC.

**Fig. 4.5** Forest plots of DOR estimates for acoustic analysis of EBNA1-IgA in the diagnosis of NPC.

**Fig. 4.6** Summary receiver operating characteristic curve of EBNA1-IgA in the diagnosis of NPC.

**Fig. 4.7** Deek’s funnel plot of EBNA1-IgA.

**Fig. 5.1** Forest plots of sensitivity estimates for acoustic analysis of Rta-IgG in the diagnosis of NPC.

**Fig. 5.2** Forest plots of specificity estimates for acoustic analysis of Rta-IgG in the diagnosis of NPC.

**Fig. 5.3** Forest plots of positive likelihood estimates for acoustic analysis of Rta-IgG in the diagnosis of NPC.

**Fig. 5.4** Forest plots of negative likelihood estimates for acoustic analysis of Rta-IgG in the diagnosis of NPC.

**Fig. 5.5** Forest plots of DOR estimates for acoustic analysis of Rta-IgG in the diagnosis of NPC.

**Fig. 5.6** Summary receiver operating characteristic curve of Rta-IgG in the diagnosis of NPC.

**Fig. 5.7** Deek’s funnel plot of Rta-IgG.

**Fig. 1.1**

**Fig. 1.2**

**Fig. 1.3**

**Fig. 1.4**

**Fig. 1.5**

**Fig. 1.6**


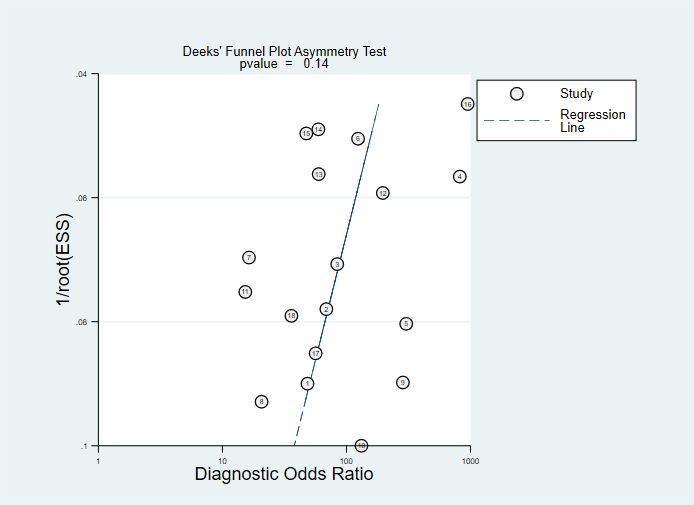


**Fig. 1.7**

**Fig. 2.1**

**Fig. 2.2**

**Fig. 2.3**

**Fig. 2.4**

**Fig. 2.5**

**Fig. 2.6**

**
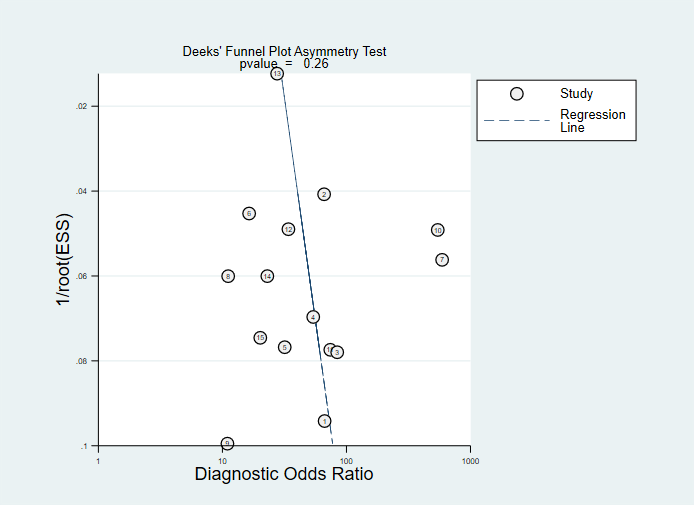
**

**Fig. 2.7**

**Fig. 3.1**

**Fig. 3.2**

**Fig. 3.3**

**Fig. 3.4**

**Fig. 3.5**

**Fig. 3.6**

**
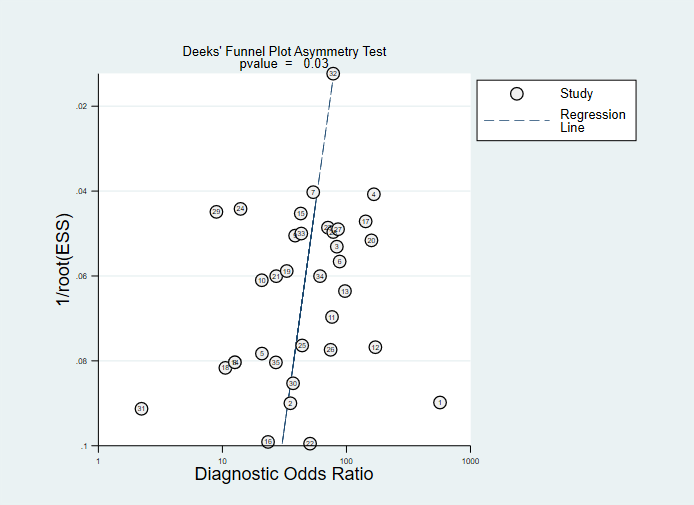
**

**Fig. 3.7**

**Fig. 4.1**

**Fig. 4.2**

**Fig. 4.3**

**Fig. 4.4**

**Fig. 4.5**

**Fig. 4.6**

**
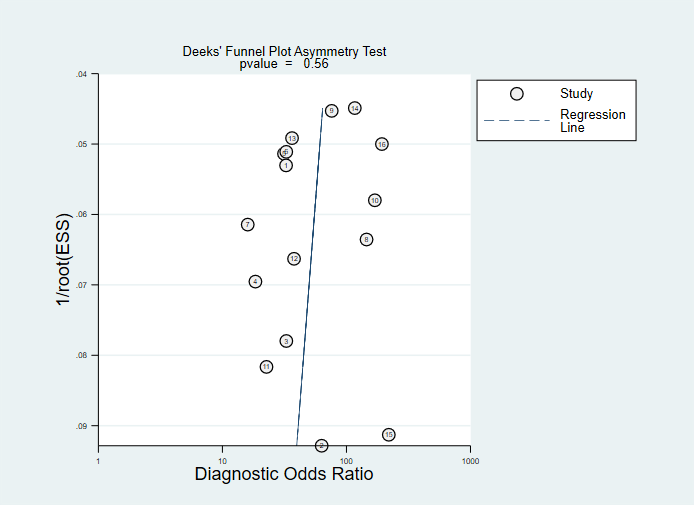
**

**Fig. 4.7**

**Fig. 5.1**

**Fig. 5.2**

**Fig. 5.3**

**Fig. 5.4**

**Fig. 5.5**

**Fig. 5.6**


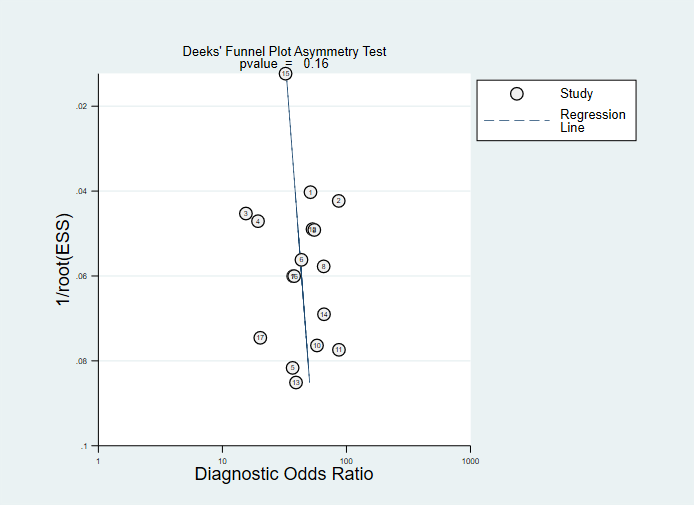


**Fig. 5.7**
